# Integrated Phytochemical, Immunological, and Network Pharmacology Investigation of Anti‐Inflammatory Lignans From *Zanthoxylum armatum* Fruits

**DOI:** 10.1002/cbdv.71806

**Published:** 2026-10-09

**Authors:** Ningthoujam Indrajit Singh, Salam Pradeep Singh, Chayan Mukherjee, Chingakham Brajakishor Singh

**Affiliations:** ^1^ BRIC‐Institute of Bioresources and Sustainable Development Imphal Manipur India; ^2^ BRIC‐Institute of Life Sciences Bhubaneshwar Orissa India

**Keywords:** bioassay‐guided fractionation, CD80, IL‐12, immunomodulatory activity, LC‐QTOF‐MS, network pharmacology

## Abstract

In the present study, bioactive lignans from *Zanthoxylum armatum* fruits have been investigated for their anti‐inflammatory properties and evaluated using dendritic cell‐based immunological assays and network pharmacology. The ethyl acetate fraction was subjected to bioassay‐guided fractionation, chromatography, and HPLC to isolate pure compounds, characterised by NMR, LC‐QTOF‐MS, and single‐crystal x‐ray diffraction. Biological activities were evaluated using DPPH/ABTS radical scavenging assays, albumin denaturation and haemolysis inhibition assays, and flow cytometric analysis of IL‐12p40/70 and CD80 expression in CpG‐stimulated MuTuDC1940 dendritic cells. Sesamin exhibited the strongest antioxidant activity, while sesamin and fargesin showed notable anti‐inflammatory effects. In CpG‐stimulated MuTuDC1940 cells, sesamin reduced the proportion of IL‐12‐positive cells from 63.85% in the CpG‐treated group to 52.67%, whereas fargesin (63.23%) showed little change and eudesmin (65.88%) showed a slight increase. Nevertheless, intracellular IL‐12 MFI was significantly reduced by eudesmin, sesamin, and fargesin compared with CpG stimulation (*p* < 0.01), suggesting modulation of IL‐12 expression intensity rather than a uniform reduction in the IL‐12‐positive cell population. Network pharmacology, PPI, GO, and KEGG analyses indicated involvement of PI3K–Akt, NF‐κB, JAK–STAT, toll‐like receptor, Rap1, and VEGF signalling pathways. These findings suggest these compounds may serve as potential natural therapeutics for inflammatory and immune‐mediated diseases.

## Graphical abstract

Bioassay‐guided fractionation of *Zanthoxylum armatum* fruits identifies three bioactive lignans—sesamin, fargesin, and eudesmin. The identified lignans attenuate CpG‐induced dendritic‐cell activation by reducing IL‐12 expression and CD80. Network pharmacology and molecular docking suggest involvement of multiple inflammatory signalling pathways, supporting their potential as anti‐inflammatory natural products.

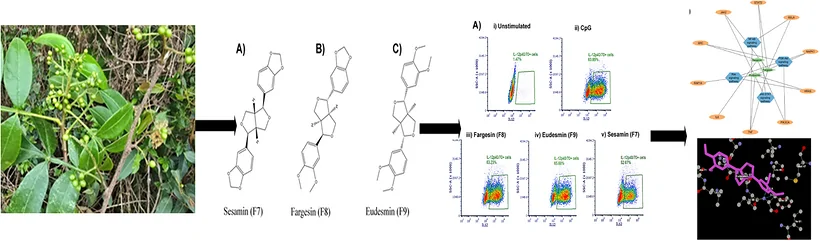

**Abbreviations**
ABTS: 2,2′‐Azino‐bis(3‐ethylbenzothiazoline‐6‐sulfonic acid)
ANOVA: Analysis of variance
BP: Biological process
CC: Cellular component
CD80: Cluster of differentiation 80
cDC1: Conventional type 1 dendritic cell
CpG: Cytosine–phosphate–guanine
DC: Dendritic cell
DPPH: 2,2‐Diphenyl‐1‐picrylhydrazyl
eGFP: Enhanced green fluorescent protein
FACS: Fluorescence‐activated cell sorting
FBS: Fetal bovine serum
GFP: Green fluorescent protein
GO: Gene Ontology
HPLC: High‐performance liquid chromatography
IBSD: Institute of Bioresources and Sustainable Development
IC_50_
: Half‐maximal inhibitory concentration
IL‐12: Interleukin‐12
IMDM: Iscove's Modified Dulbecco's Medium
JAK–STAT: Janus kinase–signal transducer and activator of transcription
KEGG: Kyoto Encyclopaedia of Genes and Genomes
LC‐QTOF‐MS: Liquid chromatography–quadrupole time‐of‐flight mass spectrometry
MAPK: Mitogen‐activated protein kinase
MCODE: Molecular Complex Detection
MF: Molecular function
MFI: Mean fluorescence intensity
MVD: Molegro Virtual Docker
NF‐κB: Nuclear factor kappa B
NMR: Nuclear magnetic resonance
NSAIDs: Non‐steroidal anti‐inflammatory drugs
PDB: Protein Data Bank
PI3K–Akt: Phosphoinositide 3‐kinase–protein kinase B signalling pathway
PKC: Protein kinase C
PPI: Protein–protein interaction
ROS: Reactive oxygen species
SD: Standard deviation
SM: Supplementary Material
SOP: Standard operating procedure
TLC: Thin‐layer chromatography
TLR: Toll‐like receptor
TLR9: Toll‐like receptor 9
VEGF: Vascular endothelial growth factor
XRD: X‐ray diffraction
ZF: Crude hydromethanolic fruit extract of *Zanthoxylum armatum*
ZFB: n‐Butanol fraction of ZF
ZFE: Ethyl acetate fraction of ZF
ZFH: Hexane fraction of ZF
ZFM: Remaining polar/methanol fraction of ZF

## 1 Introduction

Inflammation is a complicated physiological reaction of the immune system to destructive stimuli, such as pathogens, injured cells, and environmental irritants [1]. Although acute inflammation plays a critical role in host defence, chronic or uncontrolled inflammatory reactions have been implicated in the pathogenesis of many chronic diseases, such as autoimmune diseases, metabolic syndromes, cardiovascular diseases, and cancer [2]. Dendritic cells (DCs) are key regulators of innate and adaptive immunity and play an important role in inflammatory responses through antigen presentation, co‐stimulatory signalling, and cytokine production [3]. The recent progress in the field of immunology has highlighted the significance of Toll‐like receptor (TLR)‐mediated signalling in the regulation of dendritic cell activation [4]. Specifically, TLR9, when stimulated by unmethylated CpG motifs, triggers strong inflammatory responses, including co‐stimulatory molecule up‐regulation (CD80) and cytokine release (IL‐12) [5]. Though these pathways play a critical role in immune defence, activation may lead to excessive inflammation and tissue destruction. Thus, dendritic cell activation and cytokine production have been shown to be promising therapies for managing inflammatory diseases [6]. Traditional anti‐inflammatory medications, including non‐steroidal anti‐inflammatory drugs (NSAIDs), are commonly used yet are commonly linked to various adverse outcomes, which include gastrointestinal toxicity and kidney toxicity. This has led to growing interest in natural products as alternative therapeutic agents based on structural diversity, biological compatibility, and decreased side effects [7]. Consequently, increasing attention has been directed towards natural products as sources of structurally diverse bioactive compounds with potential anti‐inflammatory properties [8]. Plant‐derived secondary metabolites, especially lignans, flavonoids, and phenolic compounds, have shown important antioxidant and anti‐inflammatory effects through various molecular mechanisms, such as free radical scavenging, inhibition of pro‐inflammatory cytokines, and regulation of immune cell function. *Zanthoxylum armatum* DC has been reported to have many pharmacological uses, including antioxidant, anti‐inflammatory, anti‐microbial and anticancer [9]. Recent reviews have highlighted the broad pharmacological potential of natural products in inflammation and have emphasised the importance of identifying their molecular targets and mechanisms of action [10]. Lignans, in particular, represent a structurally diverse class of plant‐derived metabolites that have been associated with antioxidant and anti‐inflammatory activities through multiple molecular pathways, including NF‐κB‐ and oxidative‐stress‐related signalling. *Zanthoxylum armatum* DC. (Rutaceae) is a medicinally important plant that has been traditionally used for various therapeutic purposes and has been reported to possess antioxidant, anti‐inflammatory, antimicrobial, and anticancer activities [11]. Phytochemical investigations of *Z. armatum* have identified diverse secondary metabolites, including lignans, alkaloids, flavonoids, coumarins, and volatile constituents [12]. Previous pharmacological studies have also demonstrated the anti‐inflammatory activity of *Z. armatum* extracts, including its ethyl acetate fraction, and have identified several lignans, including eudesmin, fargesin, and sesamin, among its bioactive constituents [13]. However, despite the reported pharmacological potential of *Z. armatum*, the direct immunomodulatory effects of individual isolated lignans on dendritic‐cell‐mediated inflammatory responses remain insufficiently characterised. In particular, their effects in a defined TLR9/CpG‐stimulated dendritic‐cell model and their influence on IL‐12 and CD80 expression require further investigation. Moreover, integrating phytochemical characterisation with functional immunological assays and computational approaches may provide additional insight into the potential molecular mechanisms underlying the observed biological activities. In this regard, the current study aimed to isolate and characterise bioactive compounds in *Z. armatum* fruits using bioassay‐guided fractionation on the ethyl acetate fraction. The present study will provide a comprehensive evaluation of the bioactive components of *Z. armatum* and clarify their potential applications as natural anti‐inflammatory agents in dendritic cell‐mediated immune responses. This study further emphasises the potential of plant‐derived compounds as safer therapeutic agents and more effective anti‐inflammatory therapeutics by combining phytochemical characterisation with functional immunological assays.

## 2 Materials and Methods

### 2.1 Plant Material Collection and Sample Preparation

The fruits of *Z. armatum* DC. were collected from the foothills of Nongmaiching, Manipur, India (24°46′08.9″ N, 93°58′45.1″ E). Permission for plant collection was obtained from the concerned local authorities (Imphal East DC) and land owners prior to sample collection. Plant collection was conducted in accordance with institutional, local, and national regulations. The plant material was taxonomically identified and authenticated by Dr. Biseshwori Thongam, Scientist‐F, Plant Bioresources Division, BRIC‐Institute of Bioresources and Sustainable Development, Imphal, Manipur, India. A voucher specimen was deposited in the Institute's Herbarium under voucher number IBSD/M‐273 for future reference and public access. The collected fruits were washed thoroughly with water to remove impurities, shade‐dried at room temperature, and crushed into a fine powder. The powdered material was stored at 4°C.

### 2.2 Extraction and Fractionation

A total of 1.21 kg of powdered fruit material was macerated with 6 L of 70% methanol at room temperature for 6 days with intermittent stirring. The extract was filtered through Whatman filter paper, and the solvent was removed under reduced pressure on a rotary evaporator at 45°C to obtain 46 g of crude hydromethanolic extract (yield = 3.8% w/w), designated ZF. Forty grams of ZF was suspended in 250 mL of distilled water, and sequentially partitioned with 250 mL × 3 of hexane, followed by 250 mL × 3 of ethyl acetate and 250 mL × 3 of n‐butanol. The respective organic layers were combined and concentrated under reduced pressure to obtain the hexane (ZFH), ethyl acetate (ZFE), and butanol (ZFB) fractions, while the remaining polar fraction was designated as the methanol fraction (ZFM). The respective fraction yields were hexane (10 g, 25%), ethyl acetate (16 g, 40%), butanol (2 g, 5%), and methanol (9 g, 22.5%). Based on bioactivity screening, ZFE was selected for further chromatographic fractionation. The 70% methanol–water system was selected as the initial extraction solvent because its broad polarity range facilitates the extraction of both polar and moderately polar plant secondary metabolites. The crude extract was sequentially partitioned with solvents of increasing polarity to achieve preliminary separation of chemically distinct constituents. Ethyl acetate, an intermediate‐polarity solvent suitable for enrichment of several phenolic and lignan‐type constituents, yielded the bioactive ZFE fraction, which was selected for further isolation primarily on the basis of the observed antioxidant and anti‐inflammatory activities. This selection was also supported by previous reports demonstrating anti‐inflammatory activity of the ethyl acetate fraction of *Z. armatum* [9].

### 2.3 Bioassay‐Guided Isolation

The ethyl acetate fraction (ZFE; 15 g) was allowed to adsorb onto 15 g of silica gel by making a slurry in ethyl acetate and was kept overnight to let it dry completely. A glass column, measuring 100 cm in length and 5 cm in diameter, was packed with 330 g of silica gel (60–120 mesh), and ZFE‐adsorbed silica (30 g) was loaded on top of the packed column. The total solvent holding capacity of this packed column was approximately 500 mL. Elution of 500 × 3 was performed using a stepwise gradient of hexane–ethyl acetate ratio starting from 100:0, 95:5, 90:10, … up to 0: 100. Fractions of approximately 500 × 3 mL for each hexane–ethyl acetate ratio were collected and monitored by TLC using an appropriate hexane–ethyl acetate running solvent ratio. Fractions displaying similar TLC profiles were pooled to obtain 21 subfractions designated ZFE1–ZFE21. Based on antioxidant and anti‐inflammatory screening, ZFE7, ZFE8, and ZFE9 were selected for further purification. Each active subfraction was subjected to additional silica gel chromatography (100–200 mesh) using three separate columns, each 50 cm in length and 3 cm in diameter, packed with 170 g of silica gel. Elution of 150 × 3 was performed using a stepwise gradient of hexane–ethyl acetate ratio starting from 80:20, 79:21, 78:22, … up to 50:50. Fractions of approximately 150 × 3 mL for each hexane–ethyl acetate ratio were collected and monitored by TLC using an appropriate hexane–ethyl acetate running solvent ratio and fractions displaying similar TLC profiles were pooled. This procedure yielded compounds F7, F8, and F9, which were subsequently purified by preparative HPLC.

### 2.4 Antioxidant Activity Assays

The antioxidant potential of the samples was determined by evaluating their free radical‐scavenging activities using the DPPH and ABTS assays. For the DPPH assay, different concentrations of the test samples were mixed with 0.1 mM DPPH solution and incubated in the dark at room temperature for 30 min. Following incubation, the decrease in absorbance was recorded spectrophotometrically, with ascorbic acid serving as the reference standard.

For the ABTS assay, the prepared working solution was incubated with the test samples for 10 min, and then the absorbance was measured at 734 nm. The percentage radical scavenging activity was calculated for each concentration, and the IC_50_ values were determined from the corresponding dose–response inhibition curves.

### 2.5 Anti‐Inflammatory Activity Assays

For the albumin denaturation assay, samples were incubated with 1% bovine serum albumin and subjected to heat‐induced denaturation. Absorbance was recorded at 660 nm. Diclofenac was used as a reference drug. For the haemolysis inhibition assay, 10% red blood cell suspension was incubated with test samples and exposed to heat. Absorbance was measured at 560 nm, and percentage inhibition was calculated.

### 2.6 HPLC Purification and Structural Characterization

The isolated compounds were further purified using a high‐performance liquid chromatography (HPLC) system (Shimadzu LC‐20AP pumps, model nos. 02027/02028, coupled with an SPD‐20A UV–Vis detector, model no. 40766) at the Central Instrumentation Facility, Institute of Bioresources and Sustainable Development (IBSD), Imphal. Chromatographic separation was achieved using a Shimadzu C18 reversed‐phase column (10 × 250 mm, 5 µm particle size). The mobile phase consisted of methanol and water (80:20, v/v) adjusted to pH 7.0. The flow rate was maintained at 2 mL/min, and the column temperature was set at 40°C. Eluates of the major peaks were pooled and concentrated using a rotary vacuum evaporator, followed by lyophilisation (Labconco) to remove the residual solvent. Recrystallisation of the purified compounds was performed using the solvent‐layering method with ethyl acetate and dichloromethane (1:1, v/v). The purified compounds were then structurally characterised using a combination of analytical methods such as proton nuclear magnetic resonance (1H NMR), carbon nuclear magnetic resonance (13C NMR), single‐crystal x‐ray diffraction analysis and high‐resolution liquid chromatography‐quadrupole time‐of‐flight mass spectrometry (LC‐QTOF‐MS).

### 2.7 Cell Culture and Cell Viability Assessment

The murine conventional type 1 dendritic cell line MuTuDC1940 (MuTuDCs) used in this study was a kind gift from Prof. Hans Acha‐Orbea. As the cell line was obtained directly from the providing laboratory and was not purchased from a commercial cell repository, a commercial catalogue number was not applicable. The cells were maintained and handled in accordance with established laboratory standard operating procedures (SOPs) for cell culture and aseptic handling. However, specific records regarding the historical passage range, formal cell‐line authentication/identity verification, and mycoplasma‐testing status of the original cell stock were not available. MuTuDC1940 cells were cultured in IMDM supplemented with FBS and antibiotics at 37°C in a humidified 5% CO_2_ atmosphere. Cells were seeded at a density of 1 × 10^5^ cells/well and treated with sesamin, fargesin, or eudesmin (25 µg/mL), followed by stimulation with CpG oligodeoxynucleotide (1 µg/mL). MuTuDC1940 cells constitutively express GFP because they originate from CD11c:SV40LgT transgenic mice carrying an eGFP reporter. Therefore, endogenous GFP fluorescence was evaluated by flow cytometry as a proxy for relative cell viability and retention of the GFP‐positive MuTuDC population. During flow cytometric analysis, cellular debris and non‐single‐cell events were excluded before evaluation of the GFP‐positive population. No additional viability‐specific fluorescent dye was used in these experiments. Accordingly, GFP fluorescence was used as a surrogate measure of relative cell viability rather than as a conventional membrane‐integrity viability assay. Experiments were performed in three independent biological replicates (*n* = 3).

### 2.8 Flow Cytometry Analysis

MuTuDC1940 cells were pre‐treated with sesamin, fargesin, or eudesmin (25 µg/mL) for 1 h, followed by stimulation with CpG oligodeoxynucleotide (1 µg/mL) for 6 h. Brefeldin A was added during CpG stimulation to inhibit cytokine secretion and allow intracellular accumulation of IL‐12. Following stimulation, cells were collected and washed with FACS buffer. For surface staining, approximately 50 µL of the cell suspension was incubated with fluorochrome‐conjugated anti‐mouse CD80 antibody (eBioscience/Thermo Fisher Scientific, clone 16‐10A1) for 30 min at 4°C in the dark. Cells were subsequently washed with FACS buffer. For intracellular cytokine staining, cells were fixed with 2% paraformaldehyde and permeabilised using 1× permeabilisation buffer. Fixed and permeabilised cells were stained with fluorochrome‐conjugated anti‐mouse IL‐12p40/70 antibody (BD Pharmingen, Cat. No. 554480, clone C15.6) for 30 min at room temperature in the dark. Cells were then washed twice with FACS buffer to remove unbound antibody. Biological controls consisted of unstimulated MuTuDC1940 cells and CpG‐stimulated cells without compound treatment. Cellular debris and non‐single‐cell events were excluded before analysis, and GFP‐positive MuTuDC1940 cells were used for subsequent assessment of CD80 and IL‐12p40/70 expression. Samples were acquired within 24 h using a Cytek Aurora flow cytometer and analysed using FlowJo‐X software. Experiments were performed in three independent biological replicates (*n* = 3).

### 2.9 Network Pharmacology Analysis (Compound‐Related Targets)

Potential molecular targets of sesamin, fargesin, and eudesmin were predicted using SwissTargetPrediction and PharmMapper. For SwissTargetPrediction, the species was restricted to Homo sapiens, and predicted targets with a probability score ≥0.1 were retained for further analysis, resulting in 306 unique targets across the three compounds. For PharmMapper, the potential targets for each compound, ranked according to fit score ≥4.0, were selected, resulting in 280 unique targets after removal of duplicate entries. The outputs from SwissTargetPrediction and PharmMapper were subsequently combined, overlapping and duplicate targets were removed, and gene symbols were standardised using UniProt, resulting in 424 unique compound‐associated targets.

### 2.10 Collection of Inflammation‐Related Genes

Inflammation‐ and immune‐related genes were retrieved from GeneCards using the search terms “inflammation,” “inflammatory responses,” “dendritic cell activation,” and “TLR signalling.” Genes with a GeneCards relevance score ≥10 were retained. The resulting datasets comprised 219 genes for inflammation, 1000 genes for inflammatory responses, 1000 genes for dendritic‐cell activation, and 999 genes for TLR signalling. After consolidation and removal of duplicate gene entries, 1073 unique inflammation/immune‐associated genes and 1399 unique dendritic‐cell/TLR‐associated genes were obtained.

### 2.11 Identification of Overlapping Targets

The compound‐associated targets were intersected with the inflammation‐associated genes using Venny 2.1. The intersection yielded 144 common targets, which were considered candidate therapeutic targets and were subsequently used for STRING‐based protein–protein interaction (PPI) analysis and downstream GO and KEGG enrichment analyses.

### 2.12 Protein–Protein Interaction (PPI) Network Construction

To investigate the functional relationships among the predicted therapeutic targets, a PPI network was established using the STRING database. The analysis was performed with the organism limited to *Homo sapiens*, and only interactions with a confidence score ≥0.7 were retained to ensure high reliability. Proteins lacking interaction connectivity were excluded from the final network construction.

The resulting interaction network was imported into Cytoscape 3.10.3 for visualisation and topological analysis. Key network parameters, particularly degree centrality, were assessed using the CytoHubba plugin to identify highly interconnected hub genes that may play important roles in the anti‐inflammatory and immunomodulatory activities of the isolated compounds.

### 2.13 GO and KEGG Enrichment Analysis

Gene ontology (GO) functional enrichment analysis, including biological process (BP), cellular component (CC), and molecular function (MF) categories, was performed on the 144 overlapping targets. KEGG pathway enrichment analysis was performed using the DAVID database to identify significantly enriched signalling pathways [14]. Enriched GO terms and KEGG pathways with a significance threshold of *p* < 0.05 were considered statistically significant. SRplot was used to visualise the GO and KEGG enrichment results.

### 2.14 Compound–Target–Pathway Network Construction

A compound–target–pathway network was constructed to visualise the interactions among the isolated compounds, predicted therapeutic targets, and significantly enriched signalling pathways. The bioactive compounds sesamin, fargesin, and eudesmin were linked to their corresponding overlapping targets identified through network pharmacology analysis. Subsequently, significantly enriched KEGG pathways associated with the overlapping targets were integrated into the network. The interaction network was constructed and visualised using Cytoscape software (version 3.10.3). Network topological parameters, including degree centrality, were analysed to identify key regulatory targets and pathways underlying the anti‐inflammatory and immunomodulatory activities of the isolated compounds.

### 2.15 Molecular Docking Analysis

Molecular docking was performed as a supportive, hypothesis‐generating approach to investigate the potential interactions of sesamin, fargesin, and eudesmin with CD80 and IL‐12, two proteins corresponding to inflammatory/immunomodulatory endpoints examined experimentally in the CpG‐stimulated dendritic‐cell model. Three‐dimensional structures of CD80 (PDB ID: 1I8L) and IL‐12 (PDB ID: 1F42) were retrieved from the Protein Data Bank. Ligand structures were obtained from PubChem and energy minimised before docking. Docking simulations were carried out using Molegro Virtual Docker (MVD version 7.0). The docking protocol employed the selected binding pocket with default MolDock parameters. Predicted interactions were assessed based on MolDock scores, Rerank scores, hydrogen‐bonding interactions, and binding‐pose analyses. Protein–ligand interactions were subsequently visualised. The docking results were interpreted as predictive computational evidence and were not considered direct experimental confirmation of protein–ligand binding.

### 2.16 Statistical Analysis

Statistical analyses were performed using SigmaPlot 17 software (SigmaPlot Inc., USA). Cell‐free antioxidant and anti‐inflammatory assays were performed in triplicate, and the results are expressed as mean ± standard deviation (SD). Cell‐based experiments, including flow‐cytometric analysis of GFP‐positive MuTuDC1940 cells, intracellular IL‐12p40/70, and surface CD80 expression, were performed using three independent biological replicates (*n* = 3). When technical replicate measurements were obtained in an independent experiment, they were averaged to generate a single value for that biological/experimental replicate and were not treated as independent observations for statistical testing. Data distribution was assessed for normality using the Shapiro–Wilk test prior to parametric analysis. Comparisons involving more than two independent treatment groups were performed using one‐way analysis of variance (ANOVA), as a single experimental factor (treatment) was evaluated across multiple groups. When a significant overall effect was detected, Tukey's post hoc multiple‐comparison test was applied for pairwise group comparisons. A *p*‐value < 0.05 was considered statistically significant.

## 3 Results

### 3.1 Antioxidant Activity

Bioassay‐guided fractionation of the ethyl acetate fraction (ZFE) yielded 21 sub‐fractions, among which ZFE7, ZFE8, and ZFE9 demonstrated notable free radical scavenging activity (Figures S1 and S2). Among these, ZFE7 exhibited moderate antioxidant activity with IC_50_ values of 47.21 ± 0.09 µg/mL in the DPPH assay and 64.32 ± 0.08 µg/mL in the ABTS assay. Further purification of the active sub‐fraction ZFE7 isolated compound F7, which exhibited improved antioxidant activity relative to its parent fraction. Specifically, F7 demonstrated IC_50_ values of 45.72 ± 0.02 µg/mL (DPPH) and 54.27 ± 0.06 µg/mL (ABTS), as illustrated in (Figure S3). However, the antioxidant activity of F7 remained lower than that of the standard ascorbic acid (DPPH: 32.22 ± 0.14 µg/mL; ABTS: 49.30 ± 0.09 µg/mL) (Table 1). The concentration‐dependent free radical scavenging activity of all active fractions and isolated compounds is presented in detail in Tables S1 and S2.

**TABLE 1:** In vitro antioxidant activity of active sub‐fractions and isolated active phyto‐components from fruit of *Zanthoxylum armatum*.

Sample	DPPH assay IC_50_, µg/mL ± SD	ABTS assay IC_50_, µg/mL ± SD
Ascorbic acid (Control)	32.22 ± 0.14	49.30 ± 0.09
Active sub‐fractions from ethyl acetate fraction of fruit (ZFE)	Active sub‐fractions from ethyl acetate fraction of fruit (ZFE)	Active sub‐fractions from ethyl acetate fraction of fruit (ZFE)
ZFE7	47.21 ± 0.09	64.32 ± 0.08
ZFE8		
ZFE9		
ZFE7	91.41 ± 0.09	86.49 ± 0.17
ZFE8		
ZFE9		
ZFE7	50.38 ± 0.38	66.15 ± 0.04
ZFE8		
ZFE9		
Compounds isolated from ethyl acetate fraction of fruit (ZFE)	Compounds isolated from ethyl acetate fraction of fruit (ZFE)	Compounds isolated from ethyl acetate fraction of fruit (ZFE)
Sesamin (F7)	45.72 ± 0.02	54.27 ± 0.06
Fargesin (F8)		
Eudesmin (F9)		
Sesamin (F7)	85.02 ± 0.18	82.35 ± 0.04
Fargesin (F8)		
Eudesmin (F9)		
Sesamin (F7)	49.61 ± 0.16	59.45 ± 0.03
Fargesin (F8)		
Eudesmin (F9)		

NB: All values are mean ± SD (*n* = 3); ZFE7‐ active sub‐fraction number 7 from ZFE; ZFE8‐active sub‐fraction number 8 from ZFE; ZFE9 active sub‐fraction number 9 from ZFE; Isolated compound F7 = sesamin; Isolated compound F8 = fargesin; Isolated compound F9 = eudesmin.

### 3.2 Anti‐Inflammatory Activity

The anti‐inflammatory activity of the active fractions and isolated compounds was determined by haemolysis inhibition and albumin denaturation assays. Anti‐inflammatory activity was observed in ZFE7 and ZFE8 among the various fractions (Figure S4). ZFE8 was the most potent in the haemolysis assay with an IC_50_ value of 40.28 ± 0.02 µg/mL, and ZFE7 had the best activity in the albumin denaturation assay with IC_50_ value of 29.74 ± 0.25 µg/mL (Table S3). Anti‐inflammatory activity was found to be still significant after further purification of compounds F7 and F8 (Figure S5). F8 demonstrated the highest haemolysis inhibition (IC_50_: 40.22 ± 0.01 µg/mL), whereas F7 showed the strongest inhibition of protein denaturation (IC_50_: 29.43 ± 0.44 µg/mL), as summarised in Table S4. Both compounds exhibited comparable or improved activity compared with the standard drug, diclofenac (Table 2).

**TABLE 2:** In vitro anti‐inflammatory activity of active sub‐fractions and isolated active phyto‐components from fruit of *Zanthoxylum armatum*.

Sample	Heat induced haemolysis inhibition assay IC_50_, µg/mL ± SD	Heat induced albumin denaturation inhibition assay IC_50_, µg/mL ± SD
Diclofenac (Control)	58.86 ± 0.11	47.90 ± 0.15
Active sub‐fractions from ethyl acetate fraction of fruit (ZFE)	Active sub‐fractions from ethyl acetate fraction of fruit (ZFE)	Active sub‐fractions from ethyl acetate fraction of fruit (ZFE)
ZFE7	47.72 ± 0.33	29.74 ± 0.25
ZFE8		
ZFE9		
ZFE7	40.28 ± 0.02	43.65 ± 0.14
ZFE8		
ZFE9		
ZFE7	73.92 ± 0.43	46.63 ± 0.20
ZFE8		
ZFE9		
Compounds isolated from respective sub‐fractions from ethyl acetate fraction of fruit (ZFE)	Compounds isolated from respective sub‐fractions from ethyl acetate fraction of fruit (ZFE)	Compounds isolated from respective sub‐fractions from ethyl acetate fraction of fruit (ZFE)
Sesamin (F7)	46.12 ± 0.20	29.43 ± 0.44
Fargesin (F8)		
Eudesmin (F9)		
Sesamin (F7)	40.22 ± 0.01	42.78 ± 0.10
Fargesin (F8)		
Eudesmin (F9)		
Sesamin (F7)	73.96 ± 0.21	66.11 ± 0.18
Fargesin (F8)		
Eudesmin (F9)		

NB: All values are mean ± SD (*n* = 3); ZFE7‐active sub‐fraction number 7 from ZFE; ZFE8‐active sub‐fraction number 8 from ZFE; ZFE9‐active sub‐fraction number 9 from ZFE; Isolated compound F7 = sesamin; Isolated compound F8 = fargesin; Isolated compound F9 = eudesmin.

### 3.3 Structural Elucidation of Isolated Compounds

Bioassay‐guided fractionation and subsequent purification led to the isolation of three bioactive compounds, designated as F7, F8, and F9. HPLC analysis confirmed the purity and retention characteristics of the isolated compounds (see Table S5). The details of the bioactive lignans isolated from the ethyl acetate fraction of *Z. armatum* fruits and their principal biological activities are presented in Table 3. Structural elucidation identified the compounds as sesamin (F7), fargesin (F8), and eudesmin (F9). The chemical structures of the isolated compounds are presented in Figure 1.

**TABLE 3:** Bioactive lignans isolated from the ethyl acetate fraction of *Zanthoxylum armatum* fruits and their principal biological activities.

Active subfraction	Isolated compound	Identity	Major experimentally observed activity
ZFE7	F7	Sesamin	Strongest antioxidant activity among isolated compounds; strongest inhibition of albumin denaturation; prominent suppression of CpG‐induced IL‐12
ZFE8	F8	Fargesin	Strong haemolysis‐inhibitory/anti‐inflammatory activity; attenuation of dendritic‐cell activation
ZFE9	F9	Eudesmin	Moderate antioxidant and anti‐inflammatory activity; favorable predicted interactions with CD80 and IL‐12

**FIGURE 1:**
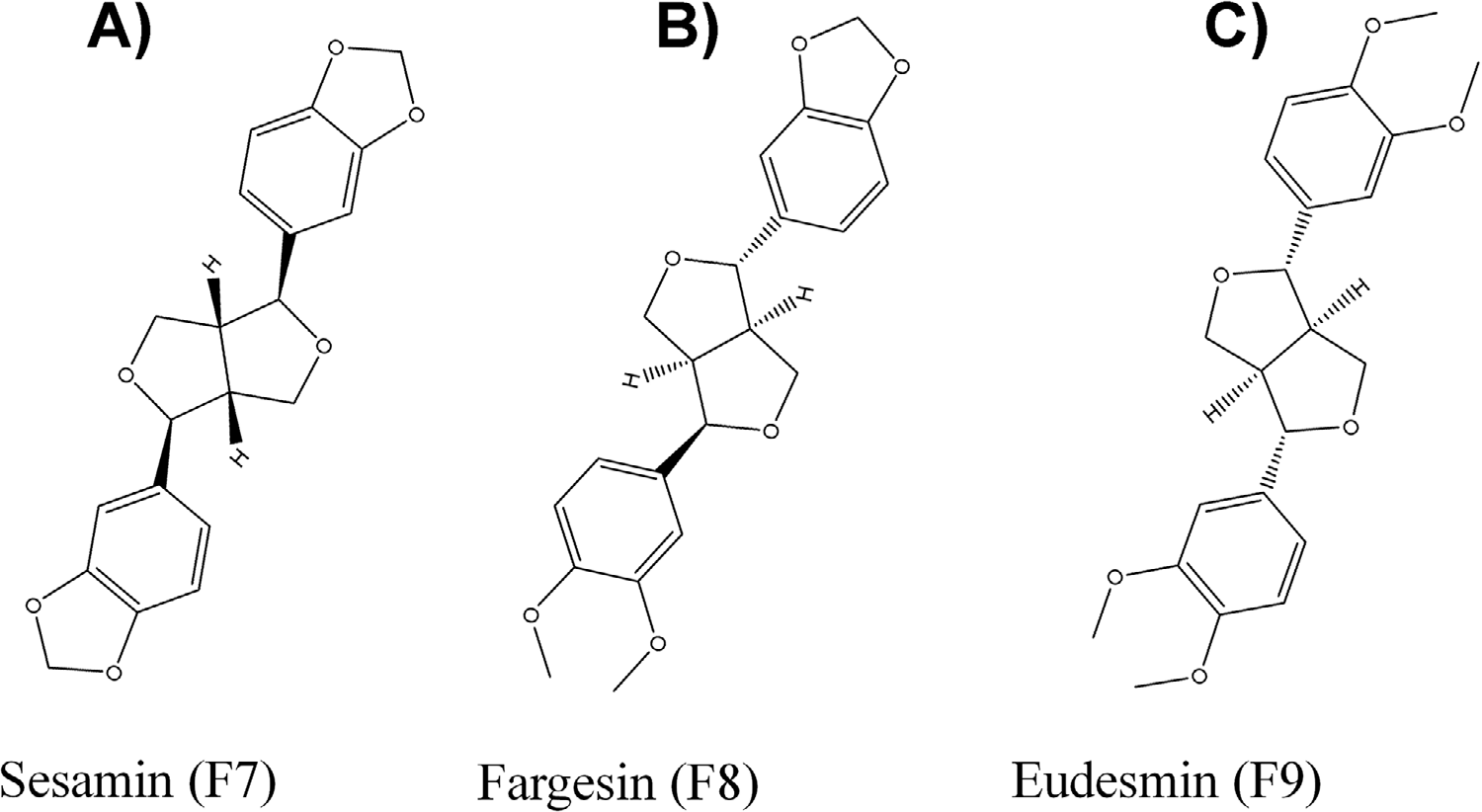
Chemical structures of the isolated bioactive compounds from *Zanthoxylum armatum*. (A) Sesamin (F7), (B) Fargesin (F8), and (C) Eudesmin (F9) isolated through bioassay‐guided fractionation of the ethyl acetate fraction of *Z. armatum* fruits. Structural elucidation was confirmed by HPLC, NMR spectroscopy, high‐resolution mass spectrometry, and single‐crystal x‐ray diffraction analysis.

The isolated compounds were characterised by single‐crystal x‐ray diffraction (XRD), and the detailed crystallographic parameters are provided in Table S6 of the SM. Spectral characterisation of the isolated compounds F7–F9 has been performed by HPLC, NMR, and mass spectrometry and is presented in Figures S6–S8. The HPLC chromatogram of compound F7 is presented in Figure S6A, which shows a narrow and well‐defined peak, which is a sign of its high purity. The ^13^C‐NMR spectrum (Figure S6B) showed characteristic carbon signals for aromatic methoxy‐substituted lignans, while the ^1^H‐NMR spectrum (Figure S6C) showed characteristic proton signals for aromatic protons, methoxy functionalities and methylenedioxy groups. In addition, the molecular ion peak in the mass spectrum (Figure S6D) was consistent with the molecular weight of sesamin, and the mass spectrum confirmed the successful identification of F7 as sesamin. Similarly, compound F8 was structurally elucidated as fargesin, through combined chromatographic and spectroscopic analysis (Figure S7). The HPLC chromatogram (Figure S7A) displayed a clear major peak with a very few impurity peaks. The ^13^C‐NMR spectrum (Figure S7B) showed typical peaks for oxygenated aromatic carbons and the methoxy‐substituted lignan skeleton, while the ^1^H‐NMR spectrum (Figure S7C) presented peaks for aromatic hydrogens and methoxy groups. The mass spectrum (Figure S7D) also showed a molecular ion peak corresponding to the expected molecular mass of fargesin, further validating the structure assignment of F8.

The isolated compound F9 was identified by spectral evidence as eudesmin (Figure S8). The HPLC chromatogram of the sample is shown in Figure 8A. The ^13^C‐NMR spectrum (Figure S8B) showed characteristic carbon resonance signals of carbon environments associated with the lignan skeleton, and the ^1^H‐NMR spectrum (Figure S8) showed proton signals. Furthermore, analysis of the mass spectrum (Figure S8D) confirmed that compound F9 was eudesmin, as evidenced by the presence of the molecular ion peak, which matches the calculated molecular weight of eudesmin. These chromatographic purity profiles, along with the NMR and mass spectral data, all confirmed the successful isolation and structural elucidation of sesamin (F7), fargesin (F8), and eudesmin (F9).

### 3.4 Inflammatory Responses in CpG‐Stimulated cDC1 Cells

The anti‐inflammatory effects of the compounds were evaluated using CpG‐stimulated MuTuDCs as an in vitro model of conventional type 1 dendritic cells (cDC1). The tested compounds did not produce a detectable reduction in the GFP‐positive MuTuDC1940 population at the applied concentration of 25 µg/mL, indicating that relative cell viability was preserved under the experimental conditions. Therefore, the observed changes in IL‐12 and CD80 expression were not attributable to an appreciable treatment‐associated reduction in the GFP‐positive cell population (Figures 2A–C and 3A–C). Flow cytometry dot plot analysis demonstrated that CpG stimulation markedly increased the proportion of IL‐12‐positive cells compared with the unstimulated control group (Figure 2A). The percentage of IL‐12‐positive cells increased from 1.47% in unstimulated cells to 63.85% following CpG stimulation. Treatment with sesamin reduced the IL‐12‐positive population to 52.67%, whereas fargesin and eudesmin showed comparatively smaller reductions, with IL‐12‐positive populations of 63.23% and 65.88%, respectively (Figure 2B).

**FIGURE 2:**
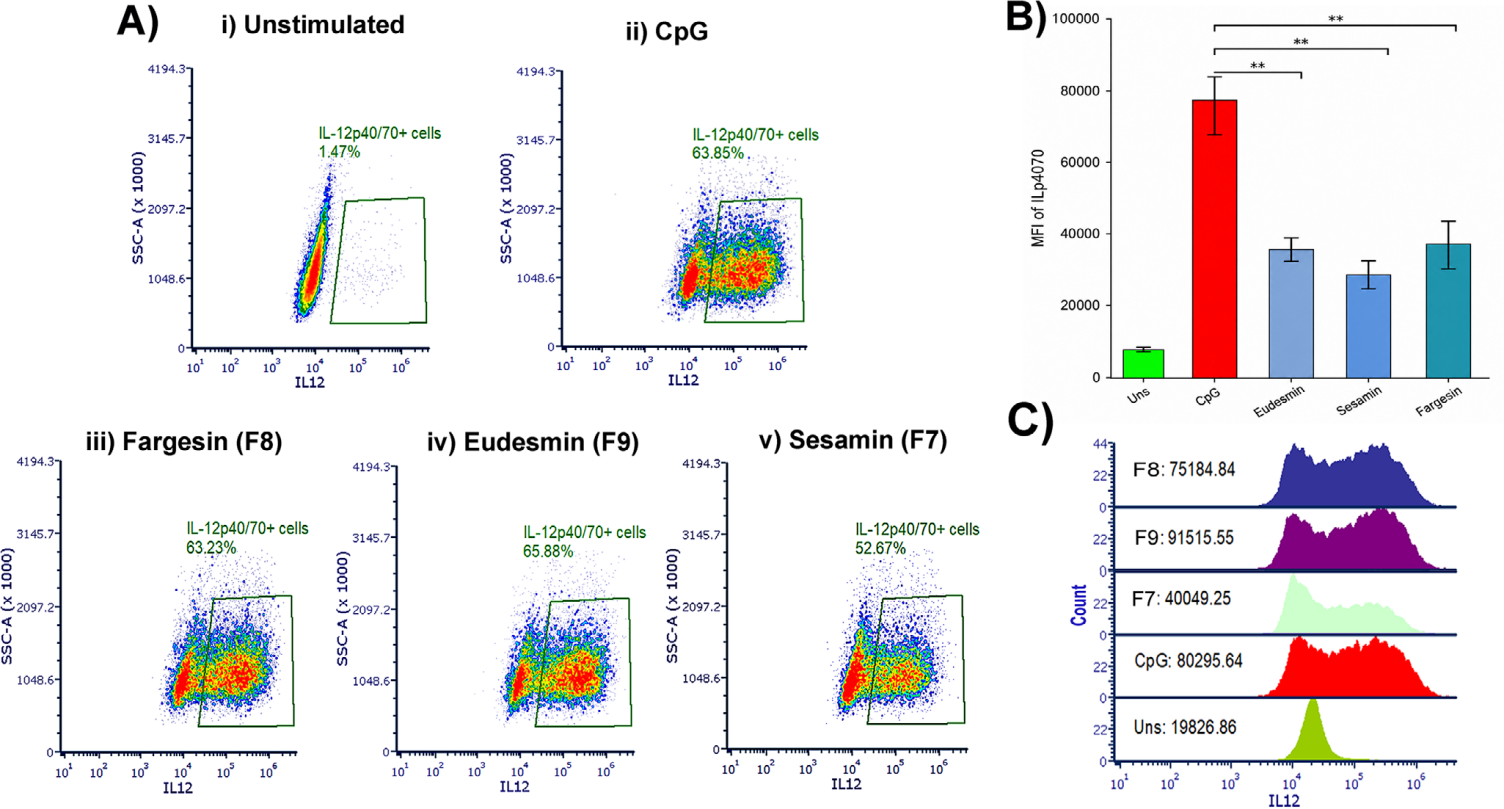
Effects of sesamin (F7), fargesin (F8), and eudesmin (F9) on intracellular IL‐12 production in CpG‐stimulated MuTuDCs. Cells were pre‐treated with the isolated compounds (25 µg/mL) for 1 h followed by stimulation with CpG oligodeoxynucleotide (1 µg/mL) for 6 h. (A) Representative flow cytometry dot plots showing intracellular IL‐12p40/70‐positive cell populations in unstimulated, CpG‐treated, and compound‐treated groups. (B) Quantitative analysis of IL‐12‐positive cell populations expressed as percentage of IL‐12‐positive cells. (C) Histogram overlay and mean fluorescence intensity (MFI) analysis of intracellular IL‐12 expression. Data are presented as mean ± SD from three independent biological experiments (*n* = 3). Statistical comparisons were performed using one‐way analysis of variance (ANOVA) followed by Tukey's post hoc multiple‐comparison test. ###*p* < 0.001 versus unstimulated control group; *p < 0.05 and **p < 0.01 versus CpG‐treated group; ns, not significant.

**FIGURE 3:**
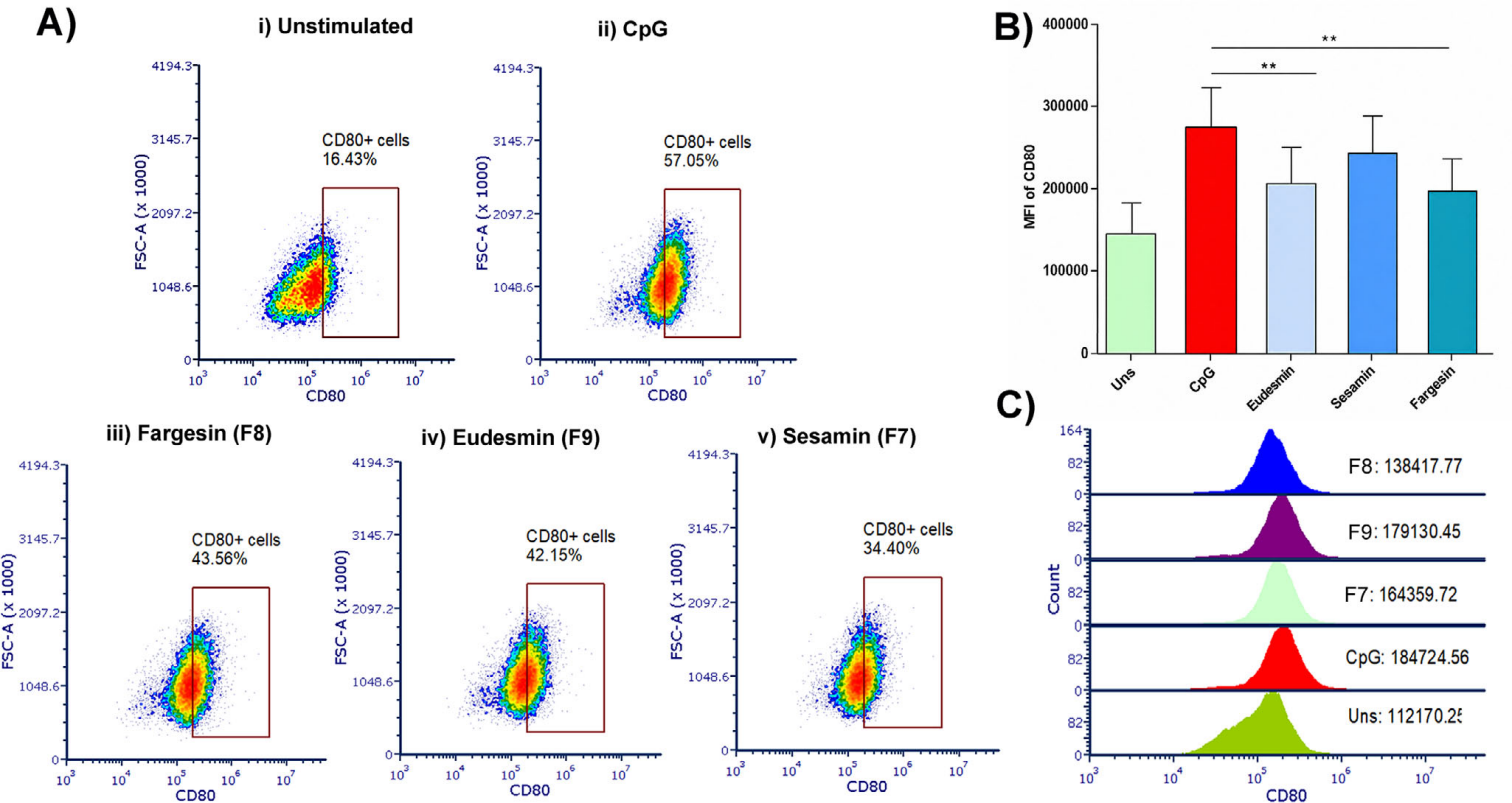
Effects of sesamin (F7), fargesin (F8), and eudesmin (F9) on CD80 expression in CpG‐stimulated MuTuDCs. (A) Flow cytometry dot plots showing CD80‐positive cell populations in unstimulated, CpG‐treated, and compound‐treated groups. (B) Quantitative analysis of CD80‐positive cell populations expressed as percentage of CD80‐positive cells. (C) Histogram overlay and mean fluorescence intensity (MFI) analysis of CD80 surface expression. Data are presented as mean ± SD from three independent biological experiments (*n* = 3). Statistical comparisons were performed using one‐way analysis of variance (ANOVA) followed by Tukey's post hoc multiple‐comparison test. ###*p* < 0.001 versus unstimulated control group; *p < 0.05 and **p < 0.01 versus CpG‐treated group; ns, not significant.

Furthermore, histogram overlay and mean fluorescence intensity (MFI) analyses were performed (Figure 2C). CpG stimulation markedly increased IL‐12 expression intensity relative to unstimulated cells. Treatment with the isolated compounds reduced IL‐12 MFI levels compared with the CpG‐treated group, indicating suppression of inflammatory cytokine production. Sesamin exhibited a greater reduction in IL‐12 expression intensity, and MFI analysis demonstrated decreased cytokine expression at the single‐cell level. Flow cytometric dot plot analysis showed that CpG stimulation substantially increased the percentage of CD80‐positive cells from 16.43% in unstimulated cells to 57.05% after CpG treatment (Figure 3A). Pre‐treatment with sesamin, fargesin, and eudesmin reduced the proportion of CD80‐positive cells to 34.40%, 43.56%, and 42.15%, respectively, indicating attenuation of dendritic cell activation (Figure 3B). Consistent with the dot plot analysis, histogram overlay and MFI quantification demonstrated that CpG markedly enhanced CD80 expression intensity, whereas treatment with the isolated compounds reduced CD80 MFI levels relative to the CpG‐treated group (Figure 3C). These findings indicate that the isolated compounds exert differential effects on CpG‐induced inflammatory responses in cDC1 cells. Sesamin reduced the proportion of IL‐12‐positive cells, while treatment with the compounds was associated with reduced intracellular IL‐12 expression intensity and attenuation of CD80 expression (Figures 2A–C and 3A–C). The effects on IL‐12‐positive cell frequency were compound‐dependent and should therefore not be interpreted as a uniform reduction by all three compounds.

### 3.5 Identification of Overlapping Targets and PPI Network Construction

The Venn analysis identified 144 common targets shared between the compound‐associated and inflammation‐related datasets, including dendritic cell/TLR‐associated targets(Figure 4A). The overlapping targets were imported into the STRING database and a PPI network was constructed which showed inflammation‐associated proteins (Figure 4B). Further, the STRING network was imported in Cytoscape and carried out for topological analysis using CytoHubba plugin which revealed key hub genes with high degree centrality (Figure 4C). The highest connectivity scores were observed for the SRC and PIK3CA genes, indicating that they are central regulators of inflammatory and immune signalling pathways. A highly interconnected functional module (MCODE score 10.364, 12 nodes, 57 edges) was also identified by the MCODE clustering analysis. The majority of the genes that were significantly enriched in this cluster were associated with receptor tyrosine kinase‐related inflammatory pathways, including SRC, JAK2, EGFR, MET, and PIK3CA. Based on these results, the isolated compounds could act as regulators of inflammatory responses by interacting with several interconnected signalling molecules involved in immune‐cell activation and cytokine production.

**FIGURE 4:**
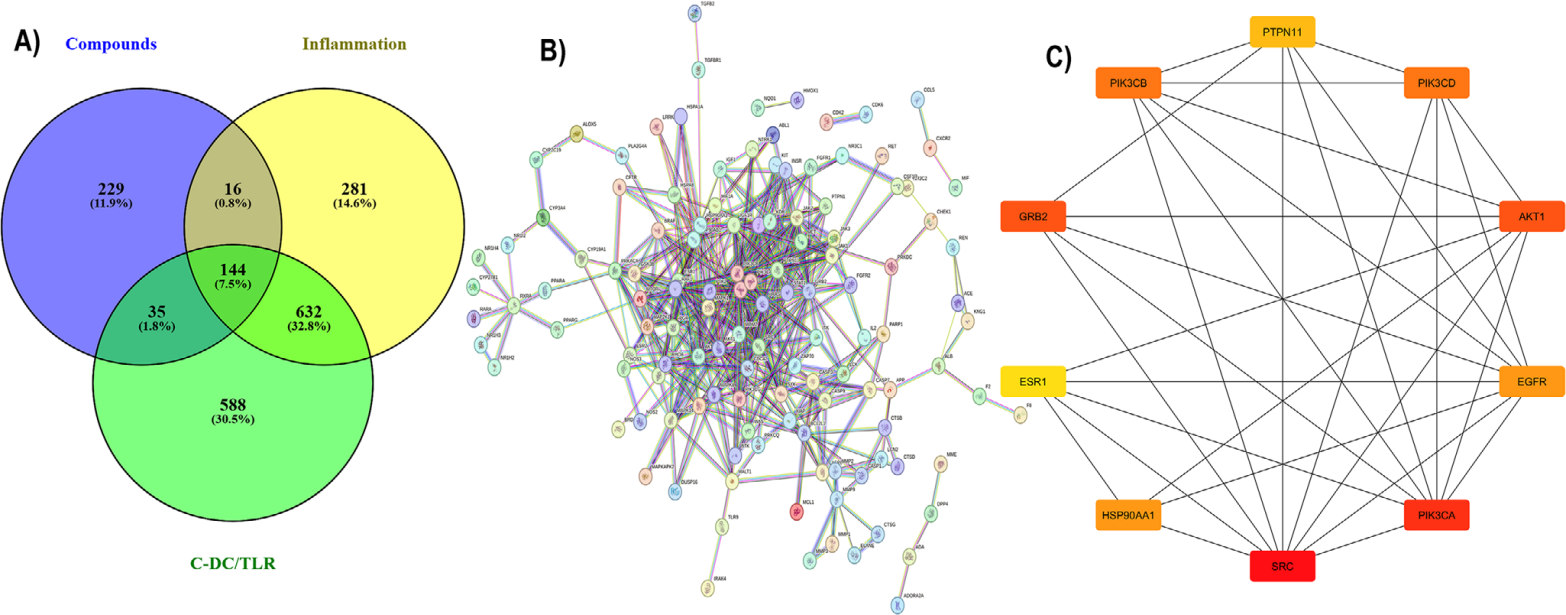
(A) Venn diagram showing overlapping targets between compound‐related targets, inflammation‐associated genes, and dendritic cell/TLR‐related genes. (B) Protein–protein interaction (PPI) network constructed using the STRING database showing interactions among overlapping therapeutic targets. (C) Identification of hub genes using CytoHubba topological analysis in Cytoscape which identified major inflammatory regulatory targets including SRC, PIK3CA, AKT1, EGFR, JAK2, and STAT3 involved in immune‐cell activation and inflammatory signaling pathways.

### 3.6 KEGG Pathway Enrichment Analysis

KEGG pathway enrichment analysis was carried out using the DAVID database to identify the major signalling pathways associated with the predicted therapeutic targets (FIgure 5A). Thus, it suggests significant enrichment of several inflammation and immune‐related pathways, including PI3K–Akt, Rap1, Ras, VEGF, focal adhesion, and EGFR‐associated signalling pathways. Among these, the PI3K–Akt pathway showed strong enrichment and involved key hub genes such as PIK3CA, AKT1, EGFR, and SRC, suggesting an important role in inflammatory and immune regulation. Enrichment of focal adhesion, Rap1, and Ras pathways indicated possible involvement in immune‐cell migration, intracellular signalling, and dendritic cell activation, whereas VEGF signalling was associated with inflammatory vascular responses. In addition, enrichment of oxidative stress‐related pathways supported the observed antioxidant activity of the isolated compounds. Overall, the enrichment results suggested that sesamin, fargesin, and eudesmin may exert anti‐inflammatory effects by regulating several related signalling pathways.

**FIGURE 5:**
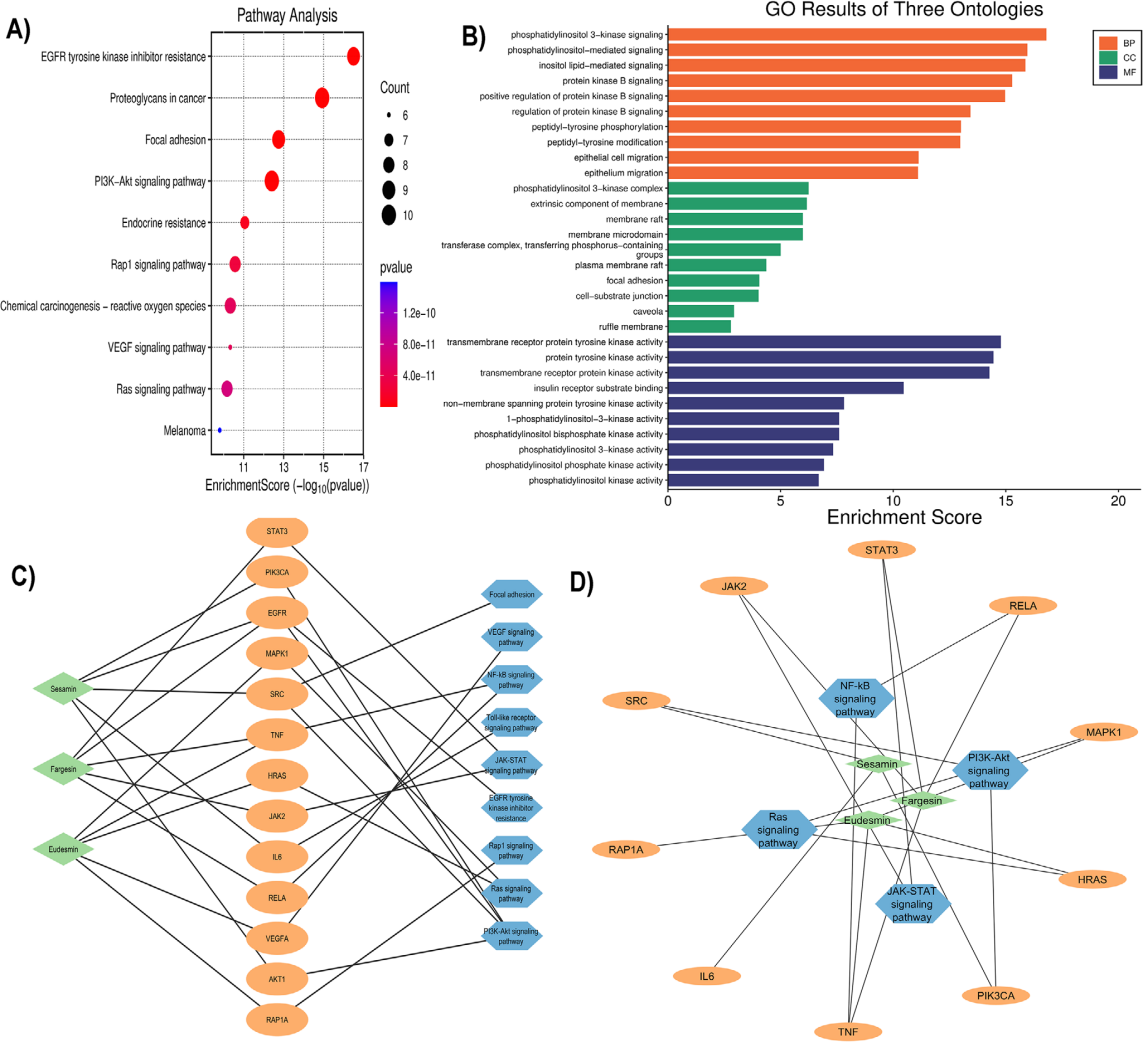
(A) KEGG pathway enrichment analysis showing significantly enriched inflammation‐ and immune‐related signaling pathways (B) Gene Ontology (GO) enrichment analysis illustrating significantly enriched biological process (BP), cellular component (CC), and molecular function (MF) categories associated with the predicted therapeutic targets. (C) Compound–target interaction network showing associations between sesamin, fargesin, eudesmin, and their predicted anti‐inflammatory target proteins. (D) Refined Compound–target–pathway network illustrating the interactions between isolated compounds, predicted therapeutic targets, and enriched KEGG signaling pathways.

### 3.7 Gene Ontology (GO) Enrichment Analysis

The overlapping targets were then subjected to GO enrichment analysis to explore the biological functions involved (Figure 5B). For the BP, most identified targets were related to PI3K‐mediated signalling, protein kinase B signalling, peptidyl‐tyrosine phosphorylation, and cell migration, suggesting their role in inflammatory signalling, immune activation, and oxidative stress response. Enrichment of the targets was mostly found in membrane rafts, membrane microdomains, focal adhesion sites, and phosphatidylinositol 3‐kinase complexes, indicating a role in membrane‐associated inflammatory signalling events. There was significant enrichment for MF, including protein tyrosine kinase activity, transmembrane receptor kinase activity, phosphatidylinositol kinase activity, and insulin receptor substrate binding. Together, these results suggest that the targets identified here are highly linked to inflammatory pathways and immune‐regulatory signalling pathways that depend on kinases.

### 3.8 Compound–Target–Pathway Network Analysis

The network analysis demonstrated that sesamin, fargesin, and eudesmin interacted with multiple inflammation‐associated targets and pathways, indicating multiple interactions among targets (Figure 5C,D). Sesamin was primarily associated with PI3K‐Akt and EGFR‐related signalling pathways through targets including AKT1, SRC, PIK3CA, and EGFR. Fargesin showed strong associations with JAK‐STAT and NF‐κB signalling pathways via JAK2, STAT3, TNF, and RELA, whereas eudesmin was primarily linked to VEGF, Ras, and Rap1 signalling pathways through VEGFA, HRAS, MAPK1, and RAP1A. The enriched pathways identified within the network included PI3K‐Akt signalling, NF‐κB signalling, JAK‐STAT signalling, TLR signalling, Ras signalling, Rap1 signalling, VEGF signalling, and focal adhesion pathways. These signalling pathways are well known to regulate dendritic cell activation, cytokine production, oxidative stress responses, and immune‐cell‐mediated inflammatory reactions. The network also highlighted SRC, PIK3CA, AKT1, EGFR, JAK2, and STAT3 as central regulatory nodes with extensive interactions among compounds and pathways, suggesting that these proteins may represent important therapeutic targets mediating the anti‐inflammatory activities of the isolated lignans. Overall, the network pharmacology analysis demonstrated that sesamin, fargesin, and eudesmin exert anti‐inflammatory and immunomodulatory effects through coordinated regulation of multiple interconnected signalling pathways involved in oxidative stress, cytokine production, dendritic cell activation, and immune signalling.

### 3.9 Molecular Docking Analysis of CD80 and IL‐12

The molecular docking results showed that all three lignans formed energetically favourable complexes with both inflammatory targets, with docking scores generally superior to those of the respective reference inhibitors. Against CD80, eudesmin showed the most favourable Rerank score (−56.72 kcal/mol). Eudesmin showed the most favourable Rerank score (−56.72), followed by sesamin (−55.24) and fargesin (−54.62), compared with −46.90 for CD80‐IN‐3 (Table 4).

**TABLE 4:** Molecular docking scores against PDB ID: 1I8L (CD80 protein).

Compound	MolDock score	Rerank score	Interaction	HBond
Eudesmin	−91.63	−56.72	−89.94	−3.46
Sesamin	−100.58	−55.24	−92.68	−2.46
Fargesin	−92.76	−54.62	−87.42	−2.39
CD80‐IN‐3 (Control)	−65.96	−46.90	−85.90	−2.50

Molecular interaction analysis indicated that eudesmin interacted with Phe59 through two oxygen‐mediated interactions, with interaction energies of −1.41 and −2.04 and distances of 3.25 and 2.85 Å, respectively (Figure 6A–C). Sesamin interacted with Ile61 and Thr62, with the Thr62–O(4) interaction showing an energy of −2.14 at a distance of 3.17 Å (Figure 6D–F).

**FIGURE 6:**
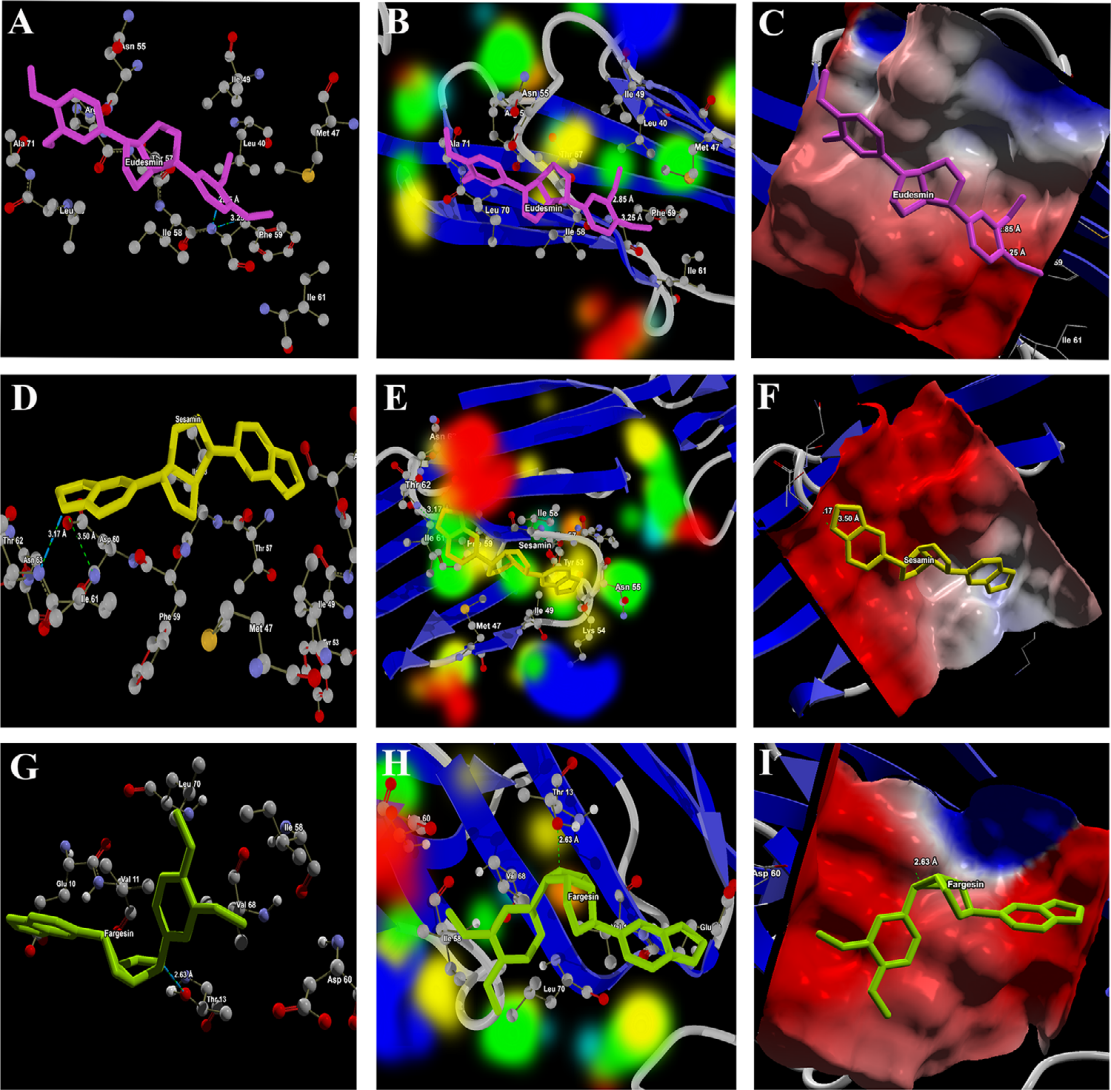
Molecular docking interactions of eudesmin, sesamin, and fargesin with CD80 (PDB ID: 1I8L). (A) Eudesmin (pink) interacted with the CD80 residue Phe59 through two oxygen atoms, O(5) and O(3), with interaction energies of −1.41 and −2.04, respectively, and interaction distances of 3.25 and 2.85 Å. (B) Energy map and (C) Electrostatic interaction map of Eudesmin at the active site of CD80.(D) Sesamin (yellow) interacted with Ile61 through O(4) at a distance of 3.50 Å with an interaction energy of −0.071, and with Thr62 through O(4) at a distance of 3.17 Å with an interaction energy of −2.14.(E) Energy map and (F) Electrostatic interaction map of Sesaminat the active site of CD80. (G) Fargesin (green) interacted with Thr13 through O(1), showing an interaction energy of −2.50 and an interaction distance of 2.63 Å.(H) Energy map and (I) Electrostatic interaction map of Fargesin at the active site of CD80.

Fargesin formed an interaction with Thr13, with an interaction energy of −2.50 and a distance of 2.63 Å (Figure 6G–I). These interactions indicate that the compounds were accommodated within the CD80 binding pocket through a combination of hydrogen‐bonding and non‐bonded interactions (Table 5). Docking analysis against IL‐12 revealed that eudesmin had the strongest predicted binding affinity, with a Rerank score of −52.84 and an interaction energy of −119.75 (Table 6). At the residue level, eudesmin interacted with Arg138 through O(4), with an interaction energy of −0.35 and a distance of 3.21 Å (Figure 7A–C). Fargesin interacted with Arg208 through O(2), with an interaction energy of −0.08 at a distance of 3.34 Å (Figure 7D–F).

**TABLE 5:** Molecular interaction analysis against PDB ID: 1I8L (CD80 protein).

Compound	Interacting residues	Interaction energy	Interaction distance
Eudesmin	Phe59—O(5)	−1.41	3.25 Å
Eudesmin	Phe59— O(3)	−2.04	2.85 Å
Sesamin	Ile61‐O(4)	−0.071	3.50 Å
Sesamin	Thr62—O(4)	−2.14	3.17 Å
Fargesin	Thr13—O(1)	−2.5	2.63 Å

**TABLE 6:** Molecular docking scores against PDB ID: 1F42 (IL‐12).

Compound	MolDock score	Rerank score	Interaction	HBond
Eudesmin	−105.62	−52.84	−119.75	−0.35
Fargesin	−79.42	−47.99	−89.24	−0.91
Sesamin	−87.90	−41.79	−95.33	−2.50
Apilimod (Control)	−49.45	−11.13	−74.13	−6.51

**FIGURE 7:**
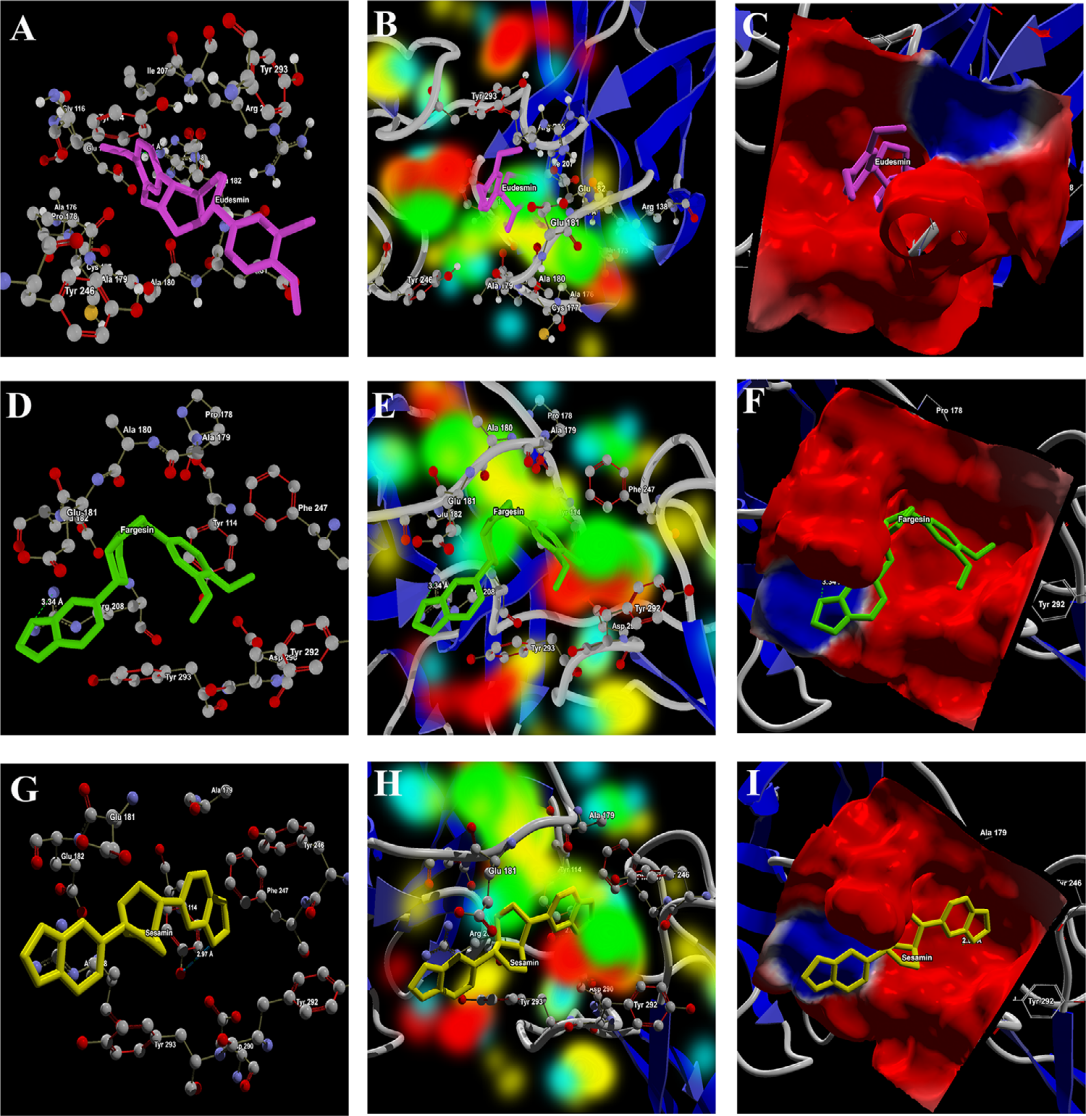
Molecular docking interactions of eudesmin, fargesin, and sesamin with IL‐12 (PDB ID: 1F42). (A) Eudesmin (pink) interacted with Arg138 through ligand atom O(4), with an interaction energy of −0.35 and an interaction distance of 3.21 Å. (B) Energy map and (C) Electrostatic interaction map of Eudesmin at the active site of IL‐12. (D) Fargesin (green) formed an interaction with Arg208 through O(2), with an interaction energy of −0.08 and an interaction distance of 3.34 Å. (E) Energy map and (F) Electrostatic interaction map of Fargesin at the active site of IL‐12. (G) Sesamin (yellow) interacted with Tyr114 through O(3), exhibiting an interaction energy of −2.50 and an interaction distance of 2.97 Å. (H) Energy map and (I) Electrostatic interaction map of Sesamin at the active site of IL‐12.

Sesamin formed a comparatively stronger interaction with Tyr114 through O(3), showing an interaction energy of −2.50 and a distance of 2.97 Å (Figure 7G–I). These residue‐level interactions support stable ligand accommodation within the IL‐12 binding region (Table 7).

**TABLE 7:** Molecular interaction analysis against PDB ID: 1F42(IL‐12).

Compound	Interacting residues	Interaction energy	Interaction distance
Eudesmin	Arg138—O(4)	−0.35	3.21 Å
Fargesin	Arg208—O(2)	−0.08	3.34 Å
Sesamin	Tyr114—O(3)	−2.5	2.97 Å

Overall, the molecular docking results identified favourable predicted interactions of sesamin, fargesin, and eudesmin with CD80 and IL‐12; however, the compound showing the most favourable predicted binding affinity was not necessarily the compound showing the strongest cellular response. In particular, eudesmin showed the most favourable Rerank scores against both CD80 and IL‐12, whereas sesamin demonstrated comparatively stronger modulation of IL‐12 expression in the cellular assay. These findings indicate that docking predictions and cellular responses provide complementary but distinct information and should not be interpreted as mutually confirmatory.

## 4 Discussion

This work combined bioassay‐guided phytochemical isolation, in vitro anti‐inflammatory assessment, immunological validation using dendritic cells and network pharmacology analysis to explore the therapeutic potential of sesamin, fargesin, and eudesmin isolated from *Z. armatum*. These results showed that these lignans have significant antioxidant, anti‐inflammatory, and immunomodulatory properties, notably by inhibiting dendritic cell activation and CpG‐induced inflammatory cytokine release. Sesamin and related lignans have also been reported to have significant anti‐inflammatory and antioxidant properties, involving modulation of oxidative stress and inflammatory signalling pathways, similar to previous studies [15]. Dendritic cells play important roles in innate and adaptive immune responses, and IL‐12 is a cytokine secreted by activated dendritic cells that significantly promotes inflammatory responses. Thus, inhibition of dendritic cell activation and IL‐12 production is a desirable therapeutic approach for inflammatory diseases. In this study, the CpG‐stimulated cDC1 model was used to simulate toll‐like receptor 9 (TLR9)‐mediated inflammatory activation, thereby enabling direct evaluation of the immunomodulatory properties of the isolated compounds [16, 17].

The antioxidant assays showed significant free radical scavenging activity, especially in sesamin‐rich fractions. Oxidative stress is linked to inflammatory responses, as the overproduction of ROS activates inflammatory signalling pathways such as NF‐κB, MAPK, and cytokine pathways. The antioxidant assays showed significant free radical scavenging activity, particularly for sesamin‐rich fractions. The DPPH and ABTS assays provide evidence of the in vitro radical‐scavenging capacity of the isolated compounds but do not directly establish their effects on intracellular oxidative stress or ROS‐dependent signalling pathways [18, 19]. Therefore, these findings are interpreted as evidence of chemical antioxidant activity. Although oxidative stress has been associated with inflammatory signalling, the present study did not directly measure intracellular ROS or oxidative‐stress‐related signalling pathways. The anti‐inflammatory and immunomodulatory effects observed in this study were instead evaluated independently using albumin denaturation and haemolysis inhibition assays and cellular flow‐cytometric analysis of IL‐12 and CD80 expression. The pharmacological relevance of the isolated compounds was further confirmed by anti‐inflammatory assays. Sesamin and fargesin showed significant anti‐denaturation and haemolysis‐inhibitory activities, suggesting that they can stabilise membrane integrity and inhibit damage to albumin (an inflammatory protein). Membrane stabilisation and inhibition of protein denaturation are well‐known anti‐inflammatory parameters that are related to the prevention of lysosomal enzyme release and injury to inflamed tissues. The isolated compounds demonstrated activities similar to those of the drug diclofenac, suggesting the therapeutic potential of these lignans as natural anti‐inflammatory agents. Previous in vitro and in vivo studies have reported similar anti‐inflammatory properties of sesamin and fargesin [20, 21].

While many phytochemical studies rely primarily on antioxidant or enzyme inhibition assays, the present work directly evaluated immune‐cell‐mediated inflammatory responses using flow cytometry. The flow‐cytometry findings indicate that the effects of the isolated lignans on IL‐12 were compound‐dependent. Although sesamin reduced the proportion of IL‐12‐positive cells from 63.85% following CpG stimulation to 52.67%, fargesin produced little change (63.23%), and eudesmin showed a slightly higher IL‐12‐positive population (65.88%). However, all three compounds significantly reduced intracellular IL‐12 MFI compared with the CpG‐treated group (*p* < 0.01), suggesting that their effects were more evident in reducing IL‐12 expression intensity within cells than in uniformly decreasing the proportion of IL‐12‐positive cells. In addition, treatment with the isolated compounds attenuated CD80 expression, suggesting inhibition of dendritic‐cell activation and co‐stimulatory signalling. Since CD80 is essential for effective T‐cell activation and amplification of adaptive immune responses, its downregulation indicates suppression of inflammatory immune activation [19].

Interestingly, differential immunomodulatory effects were observed among the isolated compounds. Sesamin demonstrated comparatively stronger suppression of IL‐12 expression intensity, whereas fargesin showed greater effects on CD80 expression.

These findings are consistent with our previous study, in which sesamin and fargesin isolated from the stem of *Z. armatum* showed anti‐inflammatory effects in CpG‐stimulated conventional type 1 dendritic cells [15]. However, the present study differs from our previous work in several important respects. First, the present investigation focuses on fruits rather than stems, representing an independent phytochemical investigation of a different plant organ. Second, the present study identified and evaluated eudesmin in addition to sesamin and fargesin, thereby expanding the chemical scope of the investigation. Third, the present work integrates the cellular findings with network pharmacology, PPI analysis, functional enrichment, compound–target–pathway network analysis, and molecular docking to investigate potential molecular mechanisms. Thus, while some experimental conditions and cellular endpoints overlap with our previous study, the present work is an independent investigation that extends the previous stem‐based findings to fruit‐derived lignans and provides additional chemical and mechanistic analyses [15].

Moreover, sesamin has been reported to suppress inflammatory mediators and oxidative stress through modulation of NF‐κB, MAPK, PI3K/Akt, and related signalling pathways in several cellular and animal models. Fargesin has also been shown to inhibit pro‐inflammatory mediators, including iNOS, COX‐2, IL‐1β, and TNF‐α, through suppression of PKC‐dependent JNK, AP‐1, and NF‐κB signalling in activated monocytes [22]. Eudesmin has likewise been reported to possess anti‐inflammatory activity, including inhibition of LPS‐induced inflammatory responses and nitric oxide production in activated immune cells. The present findings extend these observations by demonstrating that sesamin, fargesin, and eudesmin can modulate inflammatory responses in CpG‐stimulated MuTuDC1940 dendritic cells, affecting the dendritic cell activation marker CD80 and the pro‐inflammatory cytokine IL‐12. Thus, the present study provides a dendritic‐cell‐specific context for the previously reported anti‐inflammatory activities of these lignans.

Network pharmacology and enrichment analyses were used to further elucidate the molecular mechanisms underlying these biological effects. The PPI network revealed multiple hub genes such as SRC, PIK3CA, AKT1, EGFR, JAK2, and STAT3, which are known to be involved in inflammatory and immune signalling pathways. Pathway enrichment analysis using KEGG showed strong enrichment of the PI3K–Akt, NF‐κB, JAK–STAT, TLR, Ras, Rap1, VEGF, and focal adhesion signalling pathways. These pathways are closely linked to cytokine production, responses to oxidative stress, activation of immune cells and inflammatory signal transduction [23].

The enriched pathways included PI3K–Akt signalling, which is known to control the survival of DCs and their production of cytokines and inflammatory responses to TLR activation. The activation of TLR9 via CpG stimulation may lead to PI3K‐Akt‐mediated signalling pathways that stimulate the secretion of IL‐12 and maturation of DCs [24]. Thus, the decreases in IL‐12 production and CD80 expression upon treatment with sesamin and fargesin could be related to modulation of PI3K–Akt‐related inflammatory signalling. In the same way, activation of NF‐κB and JAK–STAT pathways suggests that the isolated compounds may be involved in several cytokine‐associated inflammatory pathways [25].

Enrichment of focal adhesion, Rap1, Ras, and VEGF signalling pathways further indicates possible modulation of adhesion and migration of immune cells, intracellular signalling, and inflammatory vascular response [26]. These pathways are linked to oxidative stress pathways and cytokine‐mediated activation pathways associated with chronic inflammatory diseases [27]. It is known that multi‐target pharmacological regulation is an important feature of plant‐derived bioactive compounds that play a crucial role in complex inflammatory disorders [28].

Molecular docking analysis provided additional predictive information on the potential interactions between the isolated lignans and CD80 and IL‐12. Interestingly, eudesmin showed the most favourable predicted Rerank scores against both CD80 and IL‐12, whereas sesamin demonstrated comparatively stronger modulation of IL‐12 expression in the cellular assays. This apparent discrepancy highlights the distinction between predicted ligand–protein affinity and cellular pharmacological activity [29]. Cellular responses may depend on factors beyond direct binding affinity, including compound uptake, intracellular availability, target accessibility, PPIs, and modulation of multiple signalling pathways. Therefore, the docking results should be considered complementary, hypothesis‐generating evidence rather than confirmation of the flow‐cytometry findings.

A limitation of the present network‐pharmacology analysis is the species discrepancy between the computational and experimental systems. The cellular experiments were performed using the murine MuTuDC1940 dendritic‐cell line, whereas the target prediction and downstream network analyses were based primarily on human targets. Because formal human‐to‐mouse orthologue mapping was not performed, the predicted targets and enriched pathways cannot be assumed to directly represent the corresponding molecular interactions in the murine cells. Therefore, these computational findings should be regarded as hypothesis‐generating and supportive of the experimental observations rather than definitive evidence of the molecular mechanisms operating in MuTuDC1940 cells. Future studies incorporating species‐matched target prediction, human–mouse orthologue mapping, and target‐specific experimental validation will be valuable for confirming the proposed mechanisms.

Nevertheless, molecular docking represents a predictive computational approach; therefore, direct binding assays and target‐specific functional experiments will be required to confirm the proposed interactions. It should be noted that molecular docking provides a predictive computational assessment of ligand–target interactions and does not by itself establish direct target engagement or biological causality. Although the docking results were consistent with the observed effects on CD80 expression and IL‐12 production, the predicted interactions should therefore be interpreted as supportive rather than definitive evidence of target‐specific activity. Further target‐specific experimental validation, such as direct binding assays or genetic/pharmacological target perturbation studies, will be required to confirm whether CD80 and IL‐12 are direct molecular targets of sesamin, fargesin, and eudesmin and to further establish the mechanisms underlying their immunomodulatory effects.

## 5 Conclusion

The present study successfully isolated and characterised sesamin, fargesin, and eudesmin from *Z. armatum* through bioassay‐guided fractionation. The isolated lignans exhibited significant antioxidant, anti‐inflammatory, and immunomodulatory activities, with differential effects on CpG‐induced IL‐12 responses and CD80 expression in dendritic cells. Sesamin reduced the proportion of IL‐12‐positive cells, whereas fargesin and eudesmin did not produce a corresponding reduction in this population; however, all three lignans significantly reduced intracellular IL‐12 MFI compared with CpG stimulation (*p* < 0.01), indicating modulation of IL‐12 expression intensity rather than a uniform reduction in the IL‐12‐positive cell population. Network pharmacology and enrichment analyses further suggested that these effects may involve the regulation of several inflammatory signalling pathways. Molecular docking further suggested that sesamin, fargesin, and eudesmin may interact with CD80 and IL‐12, supporting their potential involvement in the regulation of dendritic‐cell activation and cytokine‐mediated inflammatory signalling. Notably, eudesmin showed the most favourable predicted binding scores, whereas sesamin demonstrated comparatively stronger modulation of IL‐12 expression in the cellular assays, indicating that predicted binding affinity does not necessarily correspond directly to cellular efficacy. The docking results therefore provide computational support for the potential involvement of CD80 and IL‐12 in the observed immunomodulatory effects of sesamin, fargesin, and eudesmin. However, these predicted ligand–target interactions require further target‐specific experimental validation. Overall, the findings support the potential of *Z. armatum*‐derived lignans as promising natural agents for the management of dendritic‐cell‐mediated inflammatory disorders, although further mechanistic and in vivo studies are required.

## Author Contributions

**Ningthoujam Indrajit Singh**: conceptualisation, investigation, validation, methodology, writing – review & editing. **Salam Pradeep Singh**: conceptualisation, investigation, validation, methodology, writing – review & editing. **Chayan Mukherjee**: investigation, validation, methodology, writing – review & editing. **Chingakham Brajakishor Singh**: supervision, writing – original draft, methodology, writing – review & editing.

## Funding

The authors have nothing to report.

## AI Disclosure Statement

The authors declared that AI‐assisted language editing tools, such as Grammarly Edu, were used solely to improve English language quality and grammar. All scientific content was conceived and conducted by the authors. The authors accept full responsibility for the content of this manuscript.

## Ethics Statement

No new human or animal subjects were involved in this study.

## Conflicts of Interest

The authors declare no conflicts of interest.

## Supporting information




**Supporting File 1**: cbdv71806‐sup‐0001‐SuppMat.docx.

## Data Availability

All data generated or analysed during this study are included in this published article and its supplementary information files.
