# Supplementary material for: Integrated Phytochemical, Immunological, and Network Pharmacology Investigation of Anti‐Inflammatory Lignans From Zanthoxylum armatum Fruits
Source: Chem Biodivers. 2026 Oct 9;23(10):e71806. doi: 10.1002/cbdv.71806 (PMC13653114; doi:10.1002/cbdv.71806)
Supplement: Supplementary file 1 [file CBDV-23-e71806-s001.docx]

**Supplementary Material**

**Integrated Phytochemical, Immunological, and Network Pharmacology Investigation of Anti-Inflammatory Lignans from *Zanthoxylum armatum Fruits***

**Tables**

**Table ST1.** Percentage free radical scavenging activity of DPPH free radical by the three active column fractions and three isolated active phyto-components from the fruit of *Z. armatum* DC.

| Parameters (µg/mL) | Percentage free radical scavenging activity of DPPH free radical | | | | | | |
| --- | --- | --- | --- | --- | --- | --- | --- |
|  | Ascorbic acid | ZFE7 | ZFE8 | ZFE9 | F7 | F8 | F9 |
| 100 | 86.72 ± 0.16 | 53.07 ±  3.89 | 68.64 ±  0.20 | 72.13 ±  0.00 | 79.16 ±  0.83 | 71.94 ±  0.43 | 75.29 ±  1.33 |
| 80 | 86.43 ± 0.33 | 75.20 ±  0.20 | 25.20 ±  0.20 | 70.28 ±  1.02 | 76.74 ±  0.58 | 42.63 ±  0.33 | 66.95 ±  1.31 |
| 60 | 86.62 ± 0.29 | 74.18 ±  0.40 | 20.90 ±  0.40 | 65.57 ±  0.81 | 73.54 ±  0.76 | 38.27 ±  0.60 | 59.78 ±  0.16 |
| 50 | 86.53 ± 0.16 | 55.94 ±  0.20 | 14.75 ±  1.22 | 49.38 ±  0.61 | 58.62 ±  0.16 | 35.17 ±  0.76 | 50.38 ±  0.16 |
| 40 | 54.74 ± 0.16 | 34.63 ±  0.20 | 11.06 ±  0.40 | 31.35 ±  0.20 | 38.46 ±  0.16 | 30.91 ±  0.16 | 40.21 ±  0.33 |
| 20 | 42.53 ± 0.16 | 20.90 ±  1.63 | 6.96 ±  0.40 | 20.28 ±  0.20 | 33.52 ±  0.33 | 25.77 ±  1.02 | 32.17 ±  0.60 |
| 10 | 23.83 ± 1.76 | 11.06 ±  0.40 | 4.09 ±  0.40 | 14.54 ±  3.89 | 27.22 ±  0.44 | 24.03 ±  0.33 | 27.03 ±  0.87 |
| IC50 | 32.22 ± 0.14 | 47.21 ± 0.09 | 91.41 ± 0.09 | 50.38 ± 0.38 | 45.72 ± 0.02 | 85.02 ± 0.18 | 49.61 ± 0.16 |
| All values are mean ± SD (n=3); ZFE7- active sub-fraction number 7 from ZFE; ZFE8- active sub-fraction number 8 from ZFE; ZFE9- active sub-fraction number 9 from ZFE; Isolated compound F7; Isolated compound F8; Isolated compound F9. Ascorbic acid was used as positive control. | | | | | | | |

**Table ST2.** Percentage free radical scavenging activity of ABTS free radical by the three active column fractions and three isolated active phyto-components from fruit of *Z. armatum* DC.

| Parameters (µg/mL) | Percentage free radical scavenging activity of ABTS free radical | | | | | | |
| --- | --- | --- | --- | --- | --- | --- | --- |
|  | Ascorbic acid | ZFE7 | ZFE8 | ZFE9 | F7 | F8 | F9 |
| 100 | 95.21 ± 0.21 | 92.42 ±  0.42 | 68.71 ±  0.14 | 89.50 ±  0.78 | 92.78 ±  0.35 | 66.57 ±  0.14 | 93.50 ±  0.07 |
| 80 | 94.85 ± 0.28 | 76.14 ±  0.14 | 41.00 ±  0.42 | 77.21 ±  0.21 | 86.00 ±  0.28 | 47.78 ±  0.07 | 91.35 ±  0.21 |
| 60 | 79.14 ± 0.14 | 42.78 ±  0.21 | 29.92 ±  0.35 | 37.92 ±  0.21 | 65.50 ±  0.21 | 36.57 ±  0.14 | 51.35 ±  0.07 |
| 50 | 51.78 ± 0.21 | 25.35 ±  0.07 | 16.71 ±  0.71 | 20.57 ±  0.42 | 38.42 ±  0.14 | 16.42 ±  0.85 | 26.50 ±  0.50 |
| 40 | 26.00 ± 0.57 | 9.57 ±  0.85 | 11.78 ±  0.07 | 16.07 ±  0.50 | 18.50 ±  0.64 | 12.85 ±  0.71 | 21.07 ±  0.07 |
| 20 | 13.50 ± 0.64 | 3.78 ±  0.21 | 4.14 ±  0.14 | 12.42 ±  0.14 | 7.50 ±  0.50 | 7.42 ±  0.42 | 15.64 ±  0.78 |
| 10 | 10.64 ± 0.5 | -0.71 ±  0.42 | 2.00 ±  0.14 | 9.57 ±  0.14 | 1.14 ±  0.28 | 3.07 ±  1.07 | 8.85 ±  0.14 |
| IC50 | 49.30 ± 0.09 | 64.32 ± 0.08 | 86.49 ± 0.17 | 66.15 ± 0.04 | 54.27 ± 0.06 | 82.35 ± 0.04 | 59.45 ± 0.03 |
| All values are mean ± SD (n=3); ZFE7- active sub-fraction number 7 from ZFE; ZFE8- active sub-fraction number 8 from ZFE; ZFE9- active sub-fraction number 9 from ZFE; Isolated compound F7; Isolated compound F8; Isolated compound F9. Ascorbic acid was used as positive control. | | | | | | | |

**Table ST3.** Percentage inhibition of protein denaturation by the three active sub-fractions and isolated active phyto-components from fruit of *Z. armatum* DC.

| Parameters (µg/mL) | Percentage inhibition of protein denaturation | | | | | | |
| --- | --- | --- | --- | --- | --- | --- | --- |
|  | Diclofenac | ZFE7 | ZFE8 | ZFE9 | F7 | F8 | F9 |
| 100 | 69.98 ± 0.27 | 77.04 ± 0.54 | 77.25 ± 0.47 | 78.19 ± 0.82 | 80.41 ± 0.27 | 81.35 ± 0.72 | 80.88 ± 0.98 |
| 80 | 69.03 ± 0.54 | 77.09 ± 0.27 | 77.09 ± 0.54 | 73.61 ± 0.54 | 78.51 ± 1.52 | 78.35 ± 0.27 | 57.34 ± 0.47 |
| 60 | 61.13 ± 0.47 | 74.88 ± 0.94 | 73.30 ± 0.98 | 68.40 ± 1.09 | 75.19 ± 1.44 | 74.40 ± 0.82 | 46.76 ± 0.27 |
| 50 | 51.50 ± 0.27 | 74.09 ± 0.54 | 63.82 ± 0.54 | 53.08 ± 0.47 | 74.40 ± 0.47 | 66.19 ± 0.27 | 33.96 ± 0.72 |
| 40 | 44.39 ± 0.54 | 59.39 ± 0.54 | 42.02 ± 0.54 | 43.91 ± 1.09 | 58.60 ± 0.98 | 43.75 ± 0.27 | 10.74 ± 0.27 |
| 20 | 41.54 ± 0.98 | 41.07 ± 0.27 | 23.22 ± 1.70 | 28.75 ± 1.97 | 42.33 ± 0.27 | 27.17 ± 1.09 | -0.78 ± 0.54 |
| 10 | 23.85 ± 0.98 | 8.68 ± 0.98 | -2.2 ± 1.90 | 25.59 ± 0.82 | 8.68 ± 0.98 | -0.31 ± 0.27 | -4.10 ± 0.54 |
| IC50 | 47.90 ± 0.15 | 29.74 ± 0.25 | 43.65 ± 0.14 | 46.63 ± 0.20 | 29.43 ± 0.44 | 42.78 ± 0.10 | 66.11 ± 0.18 |
| All values are mean ± SD (n=3); ZFE7- active sub-fraction number 7 from ZFE; ZFE8- active sub-fraction number 8 from ZFE; ZFE9- active sub-fraction number 9 from ZFE; Isolated compound F7; Isolated compound F8; Isolated compound F9. The standard anti-inflammatory drug diclofenac was used as positive control. | | | | | | | |

**Table ST4.** Percentage inhibition of RBC membrane lysis by the three active sub-fractions and isolated active phyto-components from fruit of *Z. armatum* DC.

| Parameters (µg/mL) | Percentage inhibition of RBC membrane lysis | | | | | | |
| --- | --- | --- | --- | --- | --- | --- | --- |
|  | Diclofenac | ZFE7 | ZFE8 | ZFE9 | F7 | F8 | F9 |
| 100 | 67.46 ± 0.41 | 81.27 ± 1.49 | 66.50 ± 0.41 | 55.87 ± 0.83 | 65.78 ± 0.41 | 70.57 ± 1.24 | 56.83 ± 1.43 |
| 80 | 62.91 ± 0.41 | 70.24 ± 1.43 | 62.67 ± 0.71 | 53.95 ± 0.71 | 61.48 ± 0.41 | 63.15 ± 0.41 | 54.19 ± 0.41 |
| 60 | 51.43 ± 0.41 | 65.78 ± 0.41 | 61.48 ± 0.82 | 41.00 ± 0.71 | 57.41 ± 0.41 | 62.20 ± 0.41 | 40.28 ± 0.71 |
| 50 | 38.99 ± 1.89 | 51.43 ± 0.41 | 58.37 ± 0.71 | 32.85 ± 0.83 | 52.86 ± 0.41 | 60.28 ± 0.82 | 33.09 ± 0.71 |
| 40 | 27.51 ± 0.71 | 45.21 ± 0.41 | 49.75 ± 0.00 | 25.41 ± 1.66 | 45.45 ± 0.71 | 49.75 ± 0.00 | 24.94 ± 0.41 |
| 20 | 22.48 ± 0.71 | 37.55 ± 0.71 | 38.03 ± 0.41 | 23.02 ± 0.71 | 37.55 ± 0.71 | 38.03 ± 0.41 | 23.02 ± 0.71 |
| 10 | 20.81 ± 1.09 | 31.81 ± 2.15 | 31.81 ± 0.00 | 25.41 ± 1.66 | 31.81 ± 2.15 | 31.81 ± 0.00 | 25.41 ± 1.66 |
| IC50 | 58.86 ± 0.11 | 47.72 ± 0.33 | 40.28 ± 0.02 | 73.92 ± 0.43 | 46.12 ± 0.20 | 40.22 ± 0.01 | 73.96 ± 0.21 |
| All values are mean ± SD (n=3); ZFE7- active sub-fraction number 7 from ZFE; ZFE8- active sub-fraction number 8 from ZFE; ZFE9- active sub-fraction number 9 from ZFE; Isolated compound F7; Isolated compound F8; Isolated compound F9. The standard anti-inflammatory drug diclofenac was used as positive control. | | | | | | | |

**Table ST5.** HPLC analysis and peak table of the isolated compound

| **Sample code** | **Retention time** | **UV range** |
| --- | --- | --- |
| F7 | 13.821 | 265nm |
| F8 | 26.552 | 237nm |
| F9 | 10.063 | 289nm |

**Table ST6. Crystallographic data of isolated compounds**

| **Parameter** | **F7** | **F8** | **F9** |
| --- | --- | --- | --- |
| Formula | C20H18O6 | C21H22O6 | C22H26O6 |
| Weight | 354.34 | 370.38 | 386.43 |
| Crystal system | Monoclinic | Orthorhombic | Orthorhombic |
| Space group | P21 | P 21 21 21 | P 21 21 21 |
| a(A°) | 10.0701(11) | 5.9609(3) | 9.2563(17) |
| b(Å) | 6.9290(7) | 9.3515(6) | 12.326(3) |
| c(Å) | 11.8864(11) | 32.763(2) | 17.626(4) |
| 𝛼 (°) | 90 | 90 | 90 |
| 𝛽 (°) | 93.489(4) | 90 | 90 |
| 𝛾 (°) | 90 | 90 | 90 |
| V(Å3) | 827.84(15) | 1826.33(19) | 2011.1(7) |
| Z | 2 | 4 | 4 |
| Density (Mg m-3) | 1.422 | 1.347 | 1.276 |
| Absorption coefficient (mm-1) | 0.105 | 0.099 | 0.092 |
| F (0000) | 372 | 784 | 834 |
| Total number of reflections | 3396 | 3204 | 3537 |
| Reflections, I> 2𝜎 (1) | 2788 | 2210 | 2934 |
| Maximum 2𝜃 (°) | 26.383 | 25.027 | 25.030 |
| Ranges (h, k, l) | -12≤ h<12  -8≤ k< 8  -14≤ l< 14 | -5≤ h<7  -11≤ k<11  -39≤ l<39 | -11≤ h< 9  -14≤ k< 14  -20≤ l< 20 |
| Complete to 2(%) | 2.575 | 2.868 | 3.192 |
| Data / restraints / parameter | 3396/1/235 | 3204/0/247 | 3537/0/ 257 |
| Goodness -of-fit (F2) | 1.043 | 1.097 | 1.090 |
| Absorption correction | None | None | None |
| R indices [l> 2 𝜎(1)] | 0.1857 | 0.0939 | 0.0832 |
| R indices (all data) | 0.0852 | 0.0829 | 0.0513 |

**Supplementary Material**

**Figures**

DPPH

|  | IC50 | SD |
| --- | --- | --- |
| Bioactive column fraction (ZFE7) | 47.21 | 0.09 |
| Bioactive column fraction (ZFE8) | 91.41 | 0.09 |
| Bioactive column fraction (ZFE9) | 50.38 | 0.38 |

**SF1 Figure.** Free radical scavenging activity of three active column fractions ZFE7, ZFE8 and ZFE9 assessed byDPPH assay.

ABTS

|  | IC50 | SD |
| --- | --- | --- |
| Bioactive column fraction (ZFE7) | 64.32 | 0.08 |
| Bioactive column fraction (ZFE8) | 86.49 | 0.17 |
| Bioactive column fraction (ZFE9) | 66.14 | 0.04 |

**SF2 Figure.** Free radical scavenging activity of three active column fractions, ZFE7, ZFE8 and ZFE9 assessed by ABTS assay.

|  | DPPH | ABTS |
| --- | --- | --- |
| Abscorbic acid | 32.22 | 49.3 |
| F7 | 45.72 | 54.27 |
| F8 | 85.02 | 82.35 |
| F9 | 49.61 | 59.45 |

**SF3 Figure.** *In vitro* antioxidant activity of all isolated compounds, F7, F8 and F9, from the ethyl acetate fraction of *Z. armatum* DC fruit.

**A**

**B**

**SF4 Figure.** Anti-inflammatory activity of three active column fractions, ZFE7, ZFE8 and ZFE9 based on (A) RBC membrane lysis inhibition test. (B) Albumin anti-denaturation test.

**SF5 Figure.** *In vitro* anti-inflammatory activity of all isolated compounds, F7, F8 and F9, from the ethyl acetate fraction of *Z. armatum* DC fruit.

**SF6A Figure. Structure elucidation of isolated compound- F7- Sesamin (A) HPLC chromatogram.**

**SF6B Figure. Structure elucidation of isolated compound- F7- Sesamin (B) C-NMR.**

**SF6C Figure. Structure elucidation of isolated compound- F7- Sesamin (C) H- NMR.**

**SF6D. Figure. Structure elucidation of isolated compound- F7- Sesamin (D) Mass spectra**

**SF7A Figure. Structure elucidation of isolated compound- F8- Fargesin (A) HPLC chromatogram.**

**SF7B Figure. Structure elucidation of isolated compound- F8- Fargesin (B) C-NMR**

**SF7C Figure. Structure elucidation of isolated compound- F8- Fargesin (C) H- NMR.**

**SF7D Figure. Structure elucidation of isolated compound- F8- Fargesin (D) Mass spectra.**

**SF8A Figure. Structure elucidation of isolated compound- F9- Eudesmin (A) HPLC chromatogram.**

**SF8B. Figure. Structure elucidation of isolated compound- F9- Eudesmin (B) C-NMR.**

**SF8C. Figure. Structure elucidation of isolated compound- F9- Eudesmin (C) H- NMR.**

**SF8D. Figure. Structure elucidation of isolated compound- F9- Eudesmin (D) Mass spectra.**
